# Supplementary material for: Analysis of PPARγ Signaling Activity in Psoriasis
Source: Int J Mol Sci. 2021 Aug 10;22(16):8603. doi: 10.3390/ijms22168603 (PMC8395241; doi:10.3390/ijms22168603)
Supplement: Supplementary file 1 [file ijms-22-08603-s001.zip › Supplemental materials_Analysis of PPARg signaling activity in psoriasis/Pathway models/Models images and html files/Differentiation of psoriatic T cells/6593.html]

JAK2


# Protein JAK2

|  |  |
| --- | --- |
| URN | urn:agi-llid:3717 |
| Total Entities | 0 |
| Connectivity | 16776 |
| Name | JAK2 |
| Description | Janus kinase 2 |
| Notes | This gene product is a protein tyrosine kinase involved in a specific subset of cytokine receptor signaling pathways. It has been found to be constituitively associated with the prolactin receptor and is required for responses to gamma interferon. Mice that do not express an active protein for this gene exhibit embryonic lethality associated with the absence of definitive erythropoiesis. [provided by RefSeq, Jul 2008] |
| Primary Cell Localization | Cytoplasm |
| Class | Protein kinase |
| ObjectType | Protein |

---

|  |  |
| --- | --- |
| Pathway | Apoptosis of Cochlear Hair Cells (Noise-Induced, Mouse Model) |
|  | Leptin and CCK8 Activate Nodose Ganglia Neuron |
|  | P-cell Activation |
|  | I-cell: CCK-8 Secretion and Regulation of Eating Behavior |
|  | Serotonin Receptors Signaling |
|  | PRL and CSH Action in Mammary Gland |
|  | Thrombopoietin Receptors Signaling in Platelet Maturation |
|  | Immunoglobulin Genes Transcriptional Activation |
|  | Natural Killer Cell Activation |
|  | CD8+ T-Cell Activation |
|  | T-Cell Cytotoxic Mediated Cell Death |
|  | Th1-Cell Differentiation |
|  | AHR Signaling in Th17 Cells Function |
|  | Th17-Cell Differentiation |
|  | AHR Signaling in Tr1 Cells Function |
|  | CXC Chemokine Receptor Signaling |
|  | CC Chemokine Receptor Signaling |
|  | Proplatelet Maturation |
|  | Basophil Activation |
|  | Eosinophil Survival by Cytokine Signaling |
|  | Mast-Cell Activation via IgE Signaling |
|  | Mast-Cell Activation without Degranulation through CXCR4 Signaling |
|  | Mast-Cell Activation without Degranulation |
|  | Mast-Cell Activation without Degranulation through KIT/F2R Signaling |
|  | Macrophage M1 Lineage |
|  | CD16/CD14 Proinflammatory Monocyte Activation |
|  | AHR Signaling in M1 Macrophages Function |
|  | Neutrophil Recruitment and Priming |
|  | Negative Acute Phase Proteins Synthesis |
|  | Positive Acute Phase Proteins Synthesis |
|  | Vascular Endothelial Cell Activation by Cytokines |
|  | Vascular Endothelial Cell Activation by Blood Coagulation Factors |
|  | Vascular Endothelial Cell Activation by Growth Factors |
|  | Prolactin Induced Dopamin Synthesis by TIDA Neurons (Negative Feedback) |
|  | Adipokines Production by Adipocyte |
|  | Nociception Expression Targets Signaling |
|  | IL6 Expression Targets -> Nociception |
|  | Leptin -> NO Production/Vasodilation |
|  | Leptin -> CD25/IL6/IL10 Production |
|  | CHRNA7 -> IL8 Production |
|  | G0/G1 Phase Transition |
|  | Th1-Cells Activation and Proliferation in Atherosclerosis |
|  | Cardiomyocyte Hypertrophy |
|  | Hypertrophic Cardiomyopathy |
|  | Proteins Involved in Arterial Hypertension |
|  | TRPM4/6/7/8 Signaling in Arterial Hypertension (Hypothesis) |
|  | Proteins Involved in Myocardial Ischemia |
|  | Preconditioning Ischemia |
|  | Lymphocyte Mediated Myocardial Injury in Myocarditis |
|  | SOCS in Negative Feedback Regulation in Myocarditis |
|  | Hair Follicle Keratinocyte Apoptosis |
|  | Atopic Dermatitis |
|  | Melanoma |
|  | Apoptosis Block in Melanoma Cell |
|  | T-Cells Differentiation Block in Psoriasis |
|  | IFN-gamma/TNF-alpha Mediated Cell Proliferation in Psoriasis |
|  | Dendritic Cell Activation in Systemic Lupus Erythematosis |
|  | Th17-Cell Function in Systemic Lupus Erythematosus |
|  | Th1-Cell Function in Systemic Lupus Erythematosus |
|  | B-Cell Activation in Systemic Lupus Erythematosus |
|  | Oxidative Stress in Vitiligo |
|  | Vitiligo |
|  | T-Cell Cytotoxic Response against Melanocytes in Vitiligo |
|  | Humoral Immunity in Vitiligo |
|  | Toll-like Receptors in beta-Cell |
|  | Donohue Syndrome Progression (Hypothesis) |
|  | Tubular Cell Dysfunction in Progressive Diabetic Nephropathy |
|  | Endothelial Cell Dysfunction in Progressive Diabetic Nephropathy |
|  | Podocyte Dysfunction in Diabetic Nephropathy |
|  | Proteins Involved in Diabetic Nephropathy |
|  | Proliferative Diabetic Retinopathy |
|  | Adipokines and Cytokines in Insulin Resistance in Skeletal Muscles |
|  | Proteins Involved in Insulin Resistance |
|  | Insulin Resistance in Myocytes Induced by Obesity |
|  | Adiponectin Synthesis Declines in Insulin Resistance |
|  | Insulin Resistance in Hepatocytes |
|  | Hepatic Stellate Cells in non-Alcoholic Fatty Liver Disease |
|  | Leptin in Insulin Synthesis and Secretion |
|  | beta-Cell Destruction through Cytokines in Diabetes Mellitus |
|  | beta-Cell Mass Regulation |
|  | beta-Cell Destruction in Diabetes Mellitus Type 1 |
|  | PTPRN/STAT5 in Insulin Synthesis in beta-Cell (Rodent Model) |
|  | FOXO1 and SREBP-1C Role in beta-Cell Suppression (Rodent Model) |
|  | Hashimoto's Thyroiditis |
|  | Thyroid Hormones in Adipose Tissue Metabolism |
|  | Thyroid Dysfunction |
|  | Graves Ophthalmopathy |
|  | KISS1 Production Impairment in Hypogonadotropic hypogonadism |
|  | Hypothyroidism, Secondary (Central) |
|  | Thyroid Stimulating Hormone (TSH) Resistance in Congenital Hypothyroidism |
|  | Overt Hypothyroidism, Primary |
|  | Thyrotropin Releasing Hormone (TRH) Hypothalamic Release in non-Autoimmune Hypothyroidism |
|  | Central Hypothyroidism |
|  | Tertiary Hypothyroidism |
|  | SOCS3 and PTPN1 in Hypothalamic Neuron Insensitivity to Insulin and Leptin |
|  | Adipokines Production by Adipocyte Impaired in Obesity |
|  | CD4+T-Cell Response in Celiac Disease |
|  | Metastatic Colorectal Cancer |
|  | B-Cell Activation in Crohn's Disease |
|  | Th17-Cell Activation in Crohn's Disease |
|  | Leukocyte Migration toward the Endothelial Cell in Ulcerative Colitis |
|  | Nicotine anti-Inflammatory Effect in Ulcerative Colitis |
|  | Proteins Involved in Ulcerative Colitis |
|  | FLT3/KIT/MLL Signaling in Leukemia |
|  | Proteins Involved in Acute Myeloid Leukemia |
|  | Apoptosis Block in Acute Myeloid Leukemia Cell |
|  | RARA Signaling in Acute Myeloid Leukemia (M3) |
|  | Acute Myeloid Leukemia |
|  | B-Cell Acute Lymphoblastic Leukemia |
|  | Chronic Myeloid Leukemia |
|  | Proteins Involved in Chronic Myeloid Leukemia |
|  | Diffuse Large-B-Cell Lymphoma |
|  | Diffuse Large-B-Cell Lymphoma ABC Subtype |
|  | Primary Mediastinal B-cell Lymphoma (PMBL) Subtype |
|  | Follicular Lymphoma |
|  | Hodgkin Lymphoma |
|  | Hodgkin and Reed-Sternberg Cells Reprogramming |
|  | Mantle Cell Lymphoma |
|  | IL6/IGF1/VEGFA Signaling in Multiple Myeloma |
|  | Multiple Myeloma |
|  | Proteins Involved in T-Cell Acute Lymphoblastic Leukemia |
|  | T-Cell Acute Lymphoblastic Leukemia |
|  | Proteins Involved in Hepatocellular Carcinoma |
|  | Hepatocellular Carcinoma |
|  | Growth Factor Signaling in Hepatocellular Carcinoma |
|  | Pancreatic Ductal Carcinoma |
|  | Growth Factor Signaling in Pancreatic Neoplasms |
|  | TRPM7/8 in Pancreatic Adenocarcinoma |
|  | Eosinophil Survival in Asthma |
|  | Il17 Signaling Related Neutrophilia in Asthma |
|  | TSLP Signaling in Bronchial Epithelial Cell in Asthma |
|  | Airway Smooth Muscle Cell High Rate Proliferation |
|  | Goblet-Cell Related Mucus Secretion in Asthma |
|  | Club-Cell Role in Asthma |
|  | Basophil Activation in Asthma |
|  | Mast-Cell Activation in Asthma |
|  | Mast-Cell Related Chemokines Signaling in Asthma |
|  | Th17-Cell Differentiation in Asthma |
|  | Mucin Production in Goblet Airway Epithelial Cells |
|  | Osteoclast Activation in Gout |
|  | Neutrophil Recruitment in Sinovium in Gout |
|  | Dendritic Cell Dysfunction in Psoriatic Arthritis |
|  | Th17-Cell and Th1 Immune Responsein Psoriatic Arthritis |
|  | Osteoclast Activation in Psoriatic Arthritis |
|  | Keratinocyte Activation in Psoriatic Arthritis |
|  | Synovial Fibroblast Proliferation in Rheumatoid Arthritis |
|  | Osteoclast Activation in Rheumatoid Arthritis |
|  | Autocrine Cytokine/Chemokine Loops in Systemic Scleroderma |
|  | B-Cells Function in Systemic Scleroderma |
|  | Skin Fibrosis |
|  | CCR5 Signaling in Macrophages |
|  | Th17-Cell Numbers Reduction in HIV |
|  | CD4+ T-Cell Death in HIV |
|  | Diabetes Induced Periodontitis |
|  | Cowden Syndrome |
|  | IL6 Promotes Inflammation in Muscular Dystrophies |
|  | Bone Loss in Osteoporosis |
|  | BDNF Upregulation Triggered by Seizures |
|  | Glioma Stem Cell Program Activation |
|  | IL6/IL12 Signaling Activates Immune System in Multiple Sclerosis |
|  | Proteins Involved in Breast Cancer Related to ESR1 Signaling Pathway |
|  | Proteins Involved in Breast Cancer Related to ERBB2/VEGFR/Akt Signaling Pathway |
|  | ERBB/VEGFR/Akt Signaling in Breast Cancer |
|  | IGF1R/AKT Signaling in Breast Cancer |
|  | ESR1/ERBB Positive Luminal Breast Cancer |
|  | Proteins Involved in Breast Cancer Related to IGF1R/Akt Signaling Pathway |
|  | Breast Cancer |
|  | ESR1 Signaling in Breast Cancer |
|  | Endometrial Cancer |
|  | Endometrioid Endometrial Cancer |
|  | Clear Cell Endometrial Cancer and Papillary Serous Endometrial Cancer |
|  | Leptin Role in Endometriosis |
|  | Endometrioid Ovarian Carcinoma |
|  | Clear Cell Ovarian Carcinoma |
|  | High-Grade Serous Ovarian Carcinoma |
|  | Low-Grade Serous Ovarian Carcinoma |
|  | Ovarian Cancer |
|  | Mucinous Ovarian Carcinoma |
|  | MIF Role in Cholesteatoma |
|  | IL6/JAK/STAT3 Signaling in Cholesteatoma |
|  | Cholesteatoma |
|  | Prostate Cancer |
|  | Proteins Involved in Prostate Cancer |
|  | TLR3 Role in Retinal Pigment Epithelial Cell |
|  | Neurotrophic Factor Deprivation in Retinal Ganglion Cell Death |
|  | Proteins Involved in Noise-Induced Hearing Loss |
|  | Cochlear Inflammation (Noise-Induced) |
|  | Cytokines Trigger Otitis Media |
|  | Alveolar Macrophage Negative Regulation Declined in COPD |
|  | Mucin Hyperproduction in Goblet and Mucous Cells |
|  | Polycystins Mutation Effects in Cystic Kidney Disease |
|  | Endothelial Cell Dysfunction in Glomerulonephritis |
|  | Endothelial Cell Dysfunction in Pyelonephritis |
|  | AREG -> STAT Expression Targets |
|  | Noreadrenaline/Gq Expression Targets |
|  | Serotonin/Gq Expression Targets |
|  | Epinephrine/Gq Expression Targets |
|  | BTC -> STAT Expression Targets |
|  | IFNG -> ARRB1/STAT1 Signaling |
|  | CD22 -> STAT Expression Targets |
|  | CD72 -> NFATC Expression Targets |
|  | CD80 -> ATF/CREB/CREBBP Expression Targets |
|  | CD86 -> ATF/CREB/CREBBP Expression Targets |
|  | CLCF1 Expression Targets |
|  | CTF1 Expression Targets |
|  | CXCL12 Expression Targets |
|  | CCL3 Expression Targets |
|  | CCL3L1 Expression Target |
|  | CCL5 Expression Targets |
|  | CCL7 Expression Target |
|  | CCL7 Expression Targets |
|  | CCL8 Expression Targets |
|  | CCL15 Expression Targets |
|  | CCL2 Expression Targets |
|  | CCL4 Expression Targets |
|  | IL8 Expression Targets |
|  | CSF3 Expression Targets |
|  | CSF2 -> STAT Expression Targets |
|  | LIF Expression Targets |
|  | CNTF Expression Targets |
|  | Clozapine Induced Granulocytopenia |
|  | EGF -> STAT Expression Targets |
|  | EGFR/ERBB -> STAT Signaling |
|  | EREG -> STAT Expression Targets |
|  | Erythropoietin -> FOXO3A Expression Targets |
|  | Erythropoietin -> NF-kB Expression Targets |
|  | Erythropoietin -> AP-1/MYC/CREB Expression Targets |
|  | Erythropoietin -> STAT Expression Targets |
|  | Erythropoietin -> ELK/SRF Expression Targets |
|  | F2 -> STAT1/NF-kB Expression Targets |
|  | PLG -> STAT1/NF-kB Expression Targets |
|  | FGF2 -> STAT Expression Targets |
|  | FGF10 -> STAT Expression Targets |
|  | FGF18 -> STAT Expression Targets |
|  | FGF1 -> STAT Expression Targets |
|  | FGF9 -> STAT Expression Targets |
|  | FGF8 -> STAT Expression Targets |
|  | FGFR1 -> STAT Signaling |
|  | FGFR3 -> STAT Signaling |
|  | GH1/GHR -> NF-kB/ELK/SRF/MYC Expression Targets |
|  | GH2/GHR Expression Target |
|  | GH1/GHR -> STAT Expression Targets |
|  | CSH1/GHR Expression Targets |
|  | CSH1/PRLR Expression Targets |
|  | GH1/PRLR Expression Targets |
|  | HBEGF -> STAT Expression Targets |
|  | HGF -> STAT Expression Targets |
|  | HGFR -> STAT Signaling |
|  | Receptors and Adaptor Proteins Activated in Cancer |
|  | Genes with Mutations in Cancer-Associated Sustaining of Proliferative Signaling |
|  | G0/G1 Cell Cycle Phase Transition Activation in Cancer |
|  | STAT3 and NFkB Activate Inflammation-Induced Tumorigenesis |
|  | IL23A and IL17A Provoke Cancer-Associated Inflammation |
|  | STAT3 Inhibits Maturation of Dendritic Cells and Facilitates Cancer Progression |
|  | STAT3 Facilitates the Function of Treg Cells and Cancer Progression |
|  | IL12 Antitumor Effect is Blocked in Cancer |
|  | IL13/STAT6/ZEB1 Signalling Promotes Cancer Metastatic Progression |
|  | VEGFA Dependent Angiogenesis in Cancer |
|  | VEGF Independent Angiogenesis in Cancer |
|  | Epithelial to Mesenchymal Transition in Cancer: Overview |
|  | Hyaluronic Acid, CD44 and HMMR in Cancer Cell Invasion and Survival |
|  | Cancer Cells Inhibit Adipocyte Differentiation |
|  | Skeletal Muscle Wasting in Cancer Cachexia |
|  | Myeloid Derived Suppressor Cells in Cancer Immune Escape |
|  | IDO1 in Cancer Immune Escape |
|  | IGF2 -> STAT Expression Targets |
|  | IGF1 -> STAT Expression Targets |
|  | Insulin -> STAT Expression Targets |
|  | InsulinR -> STAT Signaling |
|  | IGF1R -> STAT Signaling |
|  | IFNG/IFNR Expression Targets |
|  | IL31R -> STAT Signaling |
|  | IL6R -> CEBP/ELK/SRF Signaling |
|  | IL3R -> STAT Signaling |
|  | IL27R -> STAT Signaling |
|  | IL5R -> STAT Signaling |
|  | IL11R -> STAT3 Signaling |
|  | IL6R -> STAT Signaling |
|  | IL12R -> STAT Signaling |
|  | IL6ST -> STAT5B Signaling |
|  | IL23R -> STAT3/NF-kB Signaling |
|  | IL12R -> NF-kB/NFATC Signaling |
|  | IL12B Expression Targets |
|  | IL3 Expression Targets |
|  | IL6 Expression Targets |
|  | IL5 Expression Targets |
|  | IL11 Expression Targets |
|  | IL31 Expression Targets |
|  | Leptin -> STAT Expression Targets |
|  | Leptin -> ELK/SRF Expression Targets |
|  | Morphine Expression Targets |
|  | NRG1 -> STAT Expression Targets |
|  | OSM/OSMR Expression Targets |
|  | OSM/IL6ST/LIFR Expression Targets |
|  | PDGFC -> STAT Expression Target |
|  | PDGFD -> STAT Expression Targets |
|  | PDGF -> STAT Expression Targets |
|  | PDGFB -> STAT Expression Target |
|  | PDGFR -> STAT Signaling |
|  | CSF2 -> STAT Signaling |
|  | KIT -> STAT Signaling |
|  | GAST Expression Targets in GAST Expression Targets |
|  | AGT -> TP53 Expression Targets |
|  | POMC Expression Targets |
|  | CCL11 Expression Targets |
|  | AGT -> CREB Expression Targets |
|  | AVP/Gq -> STAT Expression Targets |
|  | IFNA1/Gq Expression Targets |
|  | AGT -> ELK/SRF Expression Targets |
|  | AVP/Gs -> STAT Expression Targets |
|  | AGT -> STAT Expression Targets |
|  | PRL/PRLR Expression Targets |
|  | PRL/GHR -> STAT Expression Targets |
|  | PRL/GHR -> NF/kB/ELK/SRF/MYC Expression Targets |
|  | ProlactinR -> STAT Signaling |
|  | PDCD1 -> STAT Expression Targets |
|  | EphrinR -> STAT Signaling |
|  | CSF1 -> STAT Expression Targets |
|  | KITLG -> STAT Expression Targets |
|  | EphrinR Expression Targets |
|  | EFNA1 -> STAT Expression Target |
|  | PAF Expression Targets |
|  | FOXO1 Signaling |
|  | Ras-GAP Regulation Signaling |
|  | EGFR Signaling |
|  | T-Cell Receptor -> STAT Signaling |
|  | TGFA -> STAT Expression Targets |
|  | LTA Expression Targets |
|  | TNF -> STAT Expression Targets |
|  | TNFRSF1A -> STAT Signaling |
|  | Thrombopoietin -> STAT Expression Targets |
|  | ROS in Neutrophil Mediated Cell Damage |
|  | ROS in Angiotensin Mediated Cardiovascular Remodeling and Hypertrophy |
|  | B-Cell Differentiation Inhibition by Dioxin |
|  | PPAR Psoriasis |
|  | Model of PPARG signaling in psoriasis |
|  | New Pathway (3) |
|  | H4 Modification Sites |
|  | IFN signaling in alveolar cells |
|  | Dendritic Cell Activation in COVID19 |
|  | 2\_Acute phase initiation in atopic dermatitis |
|  | 1\_Differentiation of psoriatic T cells |
|  | 1\_Formation of cysts in polycystic kidney disease |
|  | 2\_The role of endothelial cells in pyelonephritis |
|  | 3\_The role of interstitial fibroblasts in pyelonephritis |
|  | 3\_Mesangial cell dysfunction in glomerulonephritis |
|  | 3\_Role of neurotrophic factor deprivation in retinal ganglion cell death |
|  | 2\_Cardiomyocyte hypertrophy |
|  | 3\_2\_Regulation of airway surface liquid (ASL) thickness |
|  | 3\_1\_Mucus accumulation in asthma |
|  | 2\_2\_Eosinophilia and neutrophilia in asthma: eosinophil apoptosis |
|  | 4\_Mucin hyperproduction in goblet and mucous cells in COPD |
|  | 1\_Defects in response to pathogens in the gut promotes inflammation in Crohn's disease |
|  | 3\_Leukocyte migration toward the endothelial cells in the intestine microvasculature |
|  | 4\_1\_Polymorphisms associated with inflammatory bowel diseases |
|  | 1\_Inflammatory mediators increase the production of hepcidin (HAMP) |
|  | 5\_The rationale for the treatment of anemia of chronic disease with erythropoietin |
|  | 4\_Inflammatory mediators inhibit GATA signaling in erythropoiesis |
|  | 3\_1\_Beta-cell proliferation and compensation: proliferation |
|  | 4\_2\_Insulin resistance: inflammation-related insulin resistance |
|  | 4\_3\_Insulin resistance: FFA-related insulin resistance |
|  | 1\_Antigen-presenting cells promote islet dysfunction and immune system activation in T1D |
|  | 3\_Islet beta-cell destruction in T1D |
|  | 1\_Decreased secretion of thyroid hormones |
|  | drug-target pairs binding |
|  | Differentiation of psoriatic T cells |

---

|  |  |
| --- | --- |
| Group | Genes with Mutations Associated with Acute Myeloid Leukemia |
|  | Genes with Mutations Associated with B-Cell Acute Lymphoblastic Leukemia |
|  | Proteins Involved in B-Cell Acute Lymphoblastic Leukemia |
|  | Genes with Mutations Associated with Diffuse Large-B-Cell Lymphoma, PMBL Subtype |
|  | Genes with Mutations Associated with Hodgkin Lymphoma |
|  | Genes with Mutations Associated with T-Cell Acute Lymphoblastic Leukemia |
|  | Proteins Involved in Cowden Syndrome |

---

|  |  |
| --- | --- |
| MedScan ID | 3717 |

---

|  |  |
| --- | --- |
| LocusLink ID | 3717 |
|  | 16452 |
|  | 24514 |
|  | 98155 |
|  | 107213 |

---

|  |  |
| --- | --- |
| Alias | OTTHUMP00000043260 |
|  | THCYT3 |
|  | Fd17 |
|  | Janus kinase II |
|  | Jak2 |
|  | Janus kinase 2 (a protein tyrosine kinase) |
|  | AI504024 |
|  | Janus tyrosine kinase 2 |
|  | C81284 |
|  | Tyrosine-protein kinase JAK2 |
|  | JAK2 kinase |
|  | JTK10 |
|  | OTTHUMP00000021024 |
|  | Janus kinase 2 |
|  | JAK-2 |
|  | C81284s |
|  | expressed sequence C81284 |
|  | expressed sequence AI504024 |

---

|  |  |
| --- | --- |
| EC Number | 2.7.10.2 |
|  | 2.7.10.1 |
|  | 2.7.1.112 |

---

|  |  |
| --- | --- |
| GO ID | 0005524 |
|  | 0042169 |
|  | 0033130 |
|  | 0005131 |
|  | 0020037 |
|  | 0042393 |
|  | 0035401 |
|  | 0043560 |
|  | 0005143 |
|  | 0046872 |
|  | 0004715 |
|  | 0051428 |
|  | 0043548 |
|  | 0008022 |
|  | 0004672 |
|  | 0019901 |
|  | 0004713 |
|  | 0005102 |
|  | 0031702 |
|  | 0007186 |
|  | 0007259 |
|  | 0060397 |
|  | 0000165 |
|  | 0030041 |
|  | 0042976 |
|  | 0000186 |
|  | 0006919 |
|  | 0097296 |
|  | 0002250 |
|  | 0006915 |
|  | 0031103 |
|  | 0007596 |
|  | 0030154 |
|  | 0019221 |
|  | 0007167 |
|  | 0030218 |
|  | 0097191 |
|  | 0060396 |
|  | 0035409 |
|  | 0060333 |
|  | 0035722 |
|  | 0038155 |
|  | 0070106 |
|  | 0070757 |
|  | 0070102 |
|  | 0035556 |
|  | 0008631 |
|  | 0061180 |
|  | 0007498 |
|  | 0001774 |
|  | 0031959 |
|  | 0050804 |
|  | 0043392 |
|  | 0010667 |
|  | 0008285 |
|  | 0022408 |
|  | 0045822 |
|  | 0043524 |
|  | 0018108 |
|  | 0048008 |
|  | 0043388 |
|  | 0051091 |
|  | 0045348 |
|  | 0046579 |
|  | 0050867 |
|  | 0045597 |
|  | 0030335 |
|  | 0010811 |
|  | 0120162 |
|  | 0007204 |
|  | 1904037 |
|  | 1902728 |
|  | 0060399 |
|  | 0050729 |
|  | 0032024 |
|  | 0050725 |
|  | 0045429 |
|  | 0051770 |
|  | 0050731 |
|  | 0014068 |
|  | 0032516 |
|  | 0010870 |
|  | 0042535 |
|  | 0032760 |
|  | 0042531 |
|  | 1904707 |
|  | 0099527 |
|  | 0046777 |
|  | 0006468 |
|  | 0046425 |
|  | 0050727 |
|  | 0060334 |
|  | 0045428 |
|  | 0046677 |
|  | 0033194 |
|  | 0070671 |
|  | 0032496 |
|  | 0034612 |
|  | 0007165 |
|  | 0033209 |
|  | 0007260 |
|  | 0005901 |
|  | 0005737 |
|  | 0005856 |
|  | 0005829 |
|  | 0031904 |
|  | 0005925 |
|  | 0098978 |
|  | 0045121 |
|  | 0016363 |
|  | 0005654 |
|  | 0005634 |
|  | 0005886 |
|  | 0098794 |
|  | 0016301 |
|  | 0000166 |
|  | 0016740 |
|  | 0071549 |
|  | 0036016 |
|  | 0071222 |
|  | 0006325 |
|  | 0009755 |
|  | 0034050 |
|  | 0002376 |
|  | 0045087 |
|  | 0030099 |
|  | 0043066 |
|  | 0060548 |
|  | 0016310 |
|  | 0043065 |
|  | 2001235 |
|  | 0008284 |
|  | 0032731 |
|  | 0045944 |
|  | 0035166 |
|  | 0097012 |
|  | 0006979 |
|  | 0016020 |
|  | 0012505 |
|  | 0033160 |
|  | 0042981 |
|  | 0005088 |
|  | 0030155 |
|  | 0005622 |
|  | 0006954 |
|  | 0031234 |
|  | 0016477 |
|  | 0007243 |
|  | 0005515 |
|  | 0038083 |
|  | 0043547 |
|  | 0042977 |
|  | 0006928 |
|  | 0016568 |
|  | 0042503 |
|  | 0042506 |
|  | 0042508 |
|  | 0016772 |
|  | 0042517 |
|  | 0007262 |
|  | 0042523 |
|  | 0042127 |
|  | 0043526 |
|  | 0005624 |
|  | 0008636 |
|  | 0051773 |
|  | 0004718 |
|  | 0023034 |
|  | 0004674 |
|  | 0000074 |
|  | 0007242 |

---

|  |  |
| --- | --- |
| KEGG ID | hsa:3717 |
|  | mmu:16452 |
|  | rno:24514 |

---

|  |  |
| --- | --- |
| MedLine Reference | 12817007 |
|  | 16828865 |
|  | 16269269 |
|  | 16847321 |
|  | 8631962 |
|  | 11493922 |
|  | 8608603 |
|  | 14565954 |
|  | 15012602 |
|  | 11201744 |
|  | 16888614 |
|  | 14982882 |
|  | 11438544 |
|  | 11821424 |
|  | 16887119 |
|  | 16959222 |
|  | 14522994 |
|  | 16230418 |
|  | 11360203 |
|  | 15522878 |
|  | 17012261 |
|  | 11440634 |
|  | 10899310 |
|  | 11940567 |
|  | 16034466 |
|  | 11799081 |
|  | 11980901 |
|  | 1848670 |
|  | 12466851 |
|  | 12351625 |
|  | 10205168 |
|  | 16930139 |
|  | 14729671 |
|  | 15948243 |
|  | 17054914 |
|  | 1281307 |
|  | 15705783 |
|  | 11940572 |
|  | 7539296 |
|  | 12626508 |
|  | 8626374 |
|  | 11217851 |
|  | 9638363 |
|  | 15927298 |
|  | 15640156 |
|  | 9154835 |
|  | 11076861 |
|  | 12370803 |
|  | 10872831 |
|  | 16373657 |
|  | 12147240 |
|  | 14551213 |
|  | 16705159 |
|  | 16741247 |
|  | 7527392 |
|  | 10198225 |
|  | 16670266 |
|  | 12426001 |
|  | 7499845 |
|  | 9343427 |
|  | 10526570 |
|  | 10918061 |
|  | 10809230 |
|  | 15016718 |
|  | 12456871 |
|  | 15121872 |
|  | 15860661 |
|  | 16174768 |
|  | 16959246 |
|  | 16572198 |
|  | 16632470 |
|  | 15530849 |
|  | 15858187 |
|  | 16557239 |
|  | 14525952 |
|  | 15958724 |
|  | 16871275 |
|  | 12106016 |
|  | 12686512 |
|  | 12478664 |
|  | 9510175 |
|  | 15604419 |
|  | 11373296 |
|  | 11114734 |
|  | 15988755 |
|  | 7530656 |
|  | 15354294 |
|  | 8876196 |
|  | 15256805 |
|  | 12244095 |
|  | 9632636 |
|  | 11694501 |
|  | 14978237 |
|  | 12107179 |
|  | 7543024 |
|  | 11591716 |
|  | 12488507 |
|  | 11881154 |
|  | 10747872 |
|  | 9553131 |
|  | 9670957 |
|  | 11971965 |
|  | 9126968 |
|  | 15815685 |
|  | 16247455 |
|  | 15169911 |
|  | 12441334 |
|  | 8657151 |
|  | 16025117 |
|  | 11698276 |
|  | 10725249 |
|  | 9624169 |
|  | 10757801 |
|  | 16478879 |
|  | 15985214 |
|  | 11294897 |
|  | 9355737 |
|  | 8611693 |
|  | 14678947 |
|  | 14551204 |
|  | 12777386 |
|  | 9618263 |
|  | 15676212 |
|  | 17105449 |
|  | 16929538 |
|  | 10635327 |
|  | 16926301 |
|  | 16084028 |
|  | 8034026 |
|  | 15591245 |
|  | 16804112 |
|  | 15716400 |
|  | 12540842 |
|  | 12223098 |
|  | 10954736 |
|  | 9590174 |
|  | 9590173 |
|  | 10922068 |
|  | 16873677 |
|  | 15677497 |
|  | 14759523 |
|  | 10064597 |
|  | 15358195 |
|  | 14996842 |
|  | 7746328 |
|  | 10842184 |
|  | 8617237 |
|  | 12594853 |
|  | 8918879 |
|  | 15932931 |
|  | 9484840 |
|  | 9326218 |
|  | 12960006 |
|  | 11042159 |
|  | 8343951 |
|  | 7607555 |
|  | 8593242 |
|  | 16091753 |
|  | 16079889 |
|  | 10531356 |
|  | 11413148 |
|  | 15790774 |
|  | 1581631 |
|  | 7540178 |
|  | 15688010 |
|  | 15615703 |
|  | 8063772 |
|  | 16204151 |
|  | 10872802 |
|  | 16923108 |
|  | 16280321 |
|  | 15701573 |
|  | 10781830 |
|  | 15688008 |
|  | 8063815 |
|  | 16514419 |
|  | 2466296 |
|  | 10925297 |
|  | 16849644 |
|  | 12007403 |
|  | 16868251 |
|  | 16420986 |
|  | 11313464 |
|  | 12244045 |
|  | 8378315 |
|  | 11163768 |
|  | 9162069 |
|  | 16728702 |
|  | 10506573 |
|  | 11839738 |
|  | 12637327 |
|  | 7889566 |
|  | 7544789 |
|  | 9473212 |
|  | 15863514 |
|  | 11944994 |
|  | 7500025 |
|  | 11818507 |
|  | 15322111 |
|  | 16620973 |
|  | 10842319 |
|  | 17078929 |
|  | 15322115 |
|  | 2482828 |
|  | 16932338 |
|  | 9689060 |
|  | 12584205 |
|  | 8912646 |
|  | 14998905 |
|  | 10026141 |
|  | 15007095 |
|  | 16365288 |
|  | 10979953 |
|  | 7534285 |
|  | 9344848 |
|  | 9446644 |
|  | 15065871 |
|  | 16442619 |
|  | 10455176 |
|  | 9548458 |
|  | 16990780 |
|  | 8208550 |
|  | 12691918 |
|  | 16257269 |
|  | 9133424 |
|  | 9573010 |
|  | 15666812 |
|  | 16757685 |
|  | 9111318 |
|  | 16772604 |
|  | 10374907 |
|  | 7535773 |
|  | 9492017 |
|  | 16567515 |
|  | 8609418 |
|  | 9452495 |
|  | 15102475 |
|  | 11585385 |
|  | 9119818 |
|  | 8943354 |
|  | 16257270 |
|  | 10551884 |
|  | 14500680 |
|  | 10224114 |
|  | 16987804 |
|  | 12921784 |
|  | 9575217 |
|  | 12456798 |
|  | 16084495 |
|  | 1620548 |
|  | 7925280 |
|  | 11430697 |
|  | 10586060 |
|  | 12393730 |
|  | 16141073 |
|  | 17057021 |
|  | 8662243 |
|  | 16141072 |
|  | 12370296 |
|  | 9169451 |
|  | 12595539 |
|  | 16572200 |
|  | 15781101 |
|  | 16990759 |
|  | 16934228 |
|  | 11593427 |
|  | 15793561 |
|  | 17024180 |
|  | 9012831 |
|  | 10502458 |
|  | 11779507 |
|  | 10200816 |
|  | 9889194 |
|  | 7613027 |
|  | 15292206 |
|  | 16502590 |
|  | 17128263 |
|  | 12952963 |
|  | 17249502 |
|  | 7690989 |
|  | 11134016 |
|  | 15923602 |
|  | 12023981 |
|  | 17077140 |
|  | 8702385 |
|  | 11934902 |
|  | 15143188 |
|  | 15837627 |
|  | 11923474 |
|  | 15143187 |
|  | 7542761 |
|  | 12748279 |
|  | 16497804 |
|  | 12777975 |
|  | 15985544 |
|  | 7775438 |
|  | 9363897 |
|  | 11679576 |
|  | 15746188 |
|  | 9727029 |
|  | 7657660 |
|  | 12023369 |
|  | 10938266 |
|  | 12920237 |
|  | 16982687 |
|  | 16705160 |
|  | 12552091 |
|  | 7812050 |
|  | 16831057 |
|  | 15611059 |
|  | 15159394 |
|  | 8756581 |
|  | 7499280 |
|  | 12538627 |
|  | 16598312 |
|  | 12477932 |
|  | 10349636 |
|  | 11731619 |
|  | 9516124 |
|  | 16959875 |
|  | 12668872 |
|  | 9837922 |
|  | 11087817 |
|  | 15156153 |
|  | 10918587 |
|  | 12576423 |
|  | 10421843 |
|  | 16598306 |
|  | 10772872 |
|  | 16603627 |
|  | 10931824 |
|  | 15899943 |
|  | 16434490 |
|  | 12941779 |
|  | 9202126 |
|  | 11152457 |
|  | 16597920 |
|  | 8995399 |
|  | 10692450 |
|  | 9342212 |
|  | 11350938 |
|  | 15198092 |
|  | 12551917 |
|  | 16885051 |
|  | 12393587 |
|  | 11875116 |
|  | 7559603 |
|  | 8934548 |
|  | 15659221 |
|  | 16877349 |
|  | 7514165 |
|  | 16914724 |
|  | 15164053 |
|  | 15805263 |
|  | 16424865 |
|  | 16537803 |
|  | 16769584 |
|  | 16904848 |
|  | 16954506 |
|  | 17045648 |
|  | 17052978 |
|  | 17068151 |
|  | 17110452 |
|  | 17111143 |
|  | 17151700 |
|  | 17169814 |
|  | 17190855 |
|  | 17198871 |
|  | 17229651 |
|  | 17229652 |
|  | 17237409 |
|  | 17263783 |
|  | 17267906 |
|  | 17296581 |
|  | 17296594 |
|  | 17315023 |
|  | 17344916 |
|  | 17356406 |
|  | 17363731 |
|  | 17376889 |
|  | 17389152 |
|  | 17403204 |
|  | 17408106 |
|  | 17426257 |
|  | 17439832 |
|  | 17440984 |
|  | 17443220 |
|  | 17460706 |
|  | 17476275 |
|  | 17488346 |
|  | 17509995 |
|  | 17596137 |
|  | 17613428 |
|  | 17625156 |
|  | 17689208 |
|  | 12388746 |
|  | 12704203 |
|  | 16563502 |
|  | 17317861 |
|  | 17519353 |
|  | 17543594 |
|  | 8022486 |
|  | 11064147 |
|  | 11244571 |
|  | 17021051 |
|  | 17332060 |
|  | 17496209 |
|  | 17526654 |

---

|  |  |
| --- | --- |
| Organism | Homo sapiens {Organism urn:agi-taxid:9606} |
|  | Mus musculus {Organism urn:agi-taxid:10090} |
|  | Rattus norvegicus {Organism urn:agi-taxid:10116} |
|  | Homo sapiens |
|  | Mus musculus |
|  | Rattus norvegicus |

---

|  |  |
| --- | --- |
| Mouse chromosome position | 19 23.73 cM |
|  | 19 24.0 cM |

---

|  |  |
| --- | --- |
| OMIM ID | 147796 |
|  | 601626 |
|  | 600880 |
|  | 133100 |
|  | 254450 |
|  | 263300 |
|  | 614521 |
|  | 187950 |

---

|  |  |
| --- | --- |
| Rat chromosome position | 1q52 |
|  | 1q51-q53 |

---

|  |  |
| --- | --- |
| Hugo ID | 6192 |
|  | HGNC:6192 |

---

|  |  |
| --- | --- |
| Human chromosome position | 9p24.1 |
|  | 9p24 |

---

|  |  |
| --- | --- |
| Swiss-Prot Accession | O60674 |
|  | A8K910 |
|  | B4DYV1 |
|  | O60674.2 |
|  | G5E852 |
|  | Q62120 |
|  | Q62120.2 |
|  | Q62689 |
|  | Q62689.1 |
|  | O14636 |
|  | O75297 |
|  | Q62124 |
|  | Q7TQD0 |
|  | Q506Q0 |
|  | Q52R86 |
|  | Q8IXP2 |
|  | Q8CIN8 |
|  | O35804 |
|  | JAK2\_RAT |
|  | JAK2\_HUMAN |
|  | JAK2\_MOUSE |

---

|  |  |
| --- | --- |
| PIR ID | JW0091 |
|  | A47511 |
|  | B39577 |
|  | JH0114 |
|  | JC4127 |

---

|  |  |
| --- | --- |
| GenBank ID | NC\_000009 |
|  | NM\_001322194 |
|  | NP\_001309123 |
|  | NM\_001322195 |
|  | NP\_001309124 |
|  | NM\_001322198 |
|  | NP\_001309127 |
|  | NM\_001322196 |
|  | NP\_001309125 |
|  | NM\_001322199 |
|  | NP\_001309128 |
|  | NM\_004972 |
|  | NP\_004963 |
|  | NM\_001322204 |
|  | NP\_001309133 |
|  | NG\_009904 |
|  | AL158147 |
|  | AL161450 |
|  | AL513350 |
|  | AY973037 |
|  | AAY16348 |
|  | CH471071 |
|  | EAW58774 |
|  | EF094945 |
|  | ABK91947 |
|  | EF194100 |
|  | ABM69254 |
|  | EU272048 |
|  | ABX71217 |
|  | KT584046 |
|  | AF001362 |
|  | AAC23653 |
|  | AF005216 |
|  | AAB82092 |
|  | AF058925 |
|  | AAC23982 |
|  | AI923337 |
|  | AK292525 |
|  | BAF85214 |
|  | AK302618 |
|  | BAG63863 |
|  | AK308668 |
|  | AY973034 |
|  | AAY22962 |
|  | BC039695 |
|  | AAH39695 |
|  | BC043187 |
|  | O60674 |
|  | NC\_000085 |
|  | XM\_006526713 |
|  | XP\_006526776 |
|  | XM\_006526715 |
|  | XP\_006526778 |
|  | NM\_008413 |
|  | NP\_032439 |
|  | NM\_001048177 |
|  | NP\_001041642 |
|  | XM\_011247155 |
|  | XP\_011245457 |
|  | XM\_017318068 |
|  | XP\_017173557 |
|  | XM\_006526714 |
|  | XP\_006526777 |
|  | AC119228 |
|  | AC162456 |
|  | AY157991 |
|  | AAN62560 |
|  | AY785782 |
|  | CH466534 |
|  | EDL41674 |
|  | AA756436 |
|  | AK087255 |
|  | BC054807 |
|  | AAH54807 |
|  | BC059834 |
|  | AAH59834 |
|  | BE981299 |
|  | BF019856 |
|  | BF730796 |
|  | BG068603 |
|  | CD363707 |
|  | CJ046359 |
|  | CJ056249 |
|  | L16956 |
|  | AAB41327 |
|  | M33423 |
|  | AAA40014 |
|  | Q62120 |
|  | NC\_005100 |
|  | NM\_031514 |
|  | NP\_113702 |
|  | AC\_000069 |
|  | AAHX01000018 |
|  | AAHX01000019 |
|  | AC109391 |
|  | CH473953 |
|  | EDM13086 |
|  | EDM13087 |
|  | AJ000557 |
|  | CAA04188 |
|  | U13396 |
|  | AAA79911 |
|  | Q62689 |
|  | AMYH02019083 |
|  | AAHY01142971 |
|  | AAHY01142970 |
|  | XM\_006231195 |
|  | AMYH02019081 |
|  | AMYH02019082 |
|  | XP\_005251512 |
|  | AAHY01142968 |
|  | AAHY01142969 |
|  | CAD13329 |
|  | XM\_005251455 |
|  | AC\_000041 |
|  | AC\_000141 |
|  | NC\_018920 |
|  | ABBA01065417 |
|  | ABBA01065418 |
|  | XP\_006231257 |
|  | AABR06010486 |
|  | AABR06010485 |
|  | AABR06010487 |
|  | NT\_008413 |
|  | NW\_001839149 |
|  | NW\_924062 |
|  | AC\_000052 |
|  | Q506Q0 |
|  | Q52R86 |
|  | Q8IXP2 |
|  | NT\_039687 |
|  | NW\_001030643 |
|  | Q8CIN8 |
|  | NW\_001084774 |
|  | NW\_047565 |
|  | O35804 |
|  | 24711711 |
|  | 409584 |
|  | 2288927 |
|  | 200572 |
|  | BG066996 |
|  | 62947930 |
|  | 2570359 |
|  | BG068848 |
|  | 595683 |
|  | 37590526 |
|  | 81866447 |
|  | 74728217 |
|  | 12643404 |
|  | NW\_000143 |
|  | Q7TQD0 |
|  | 17384438 |
|  | 2499669 |
|  | BG081632 |
|  | 2499668 |
|  | 62836431 |
|  | BG080085 |
|  | 3236322 |
|  | 3068752 |
|  | 74725766 |
|  | 32451722 |
|  | 24981011 |
|  | 81894616 |
|  | AI504024 |
|  | 74739295 |
|  | AU023352 |
|  | 119579178 |
|  | C81284 |
|  | 81886639 |
|  | 118430822 |
|  | 123187091 |
|  | 148709728 |
|  | 149062663 |
|  | 149062664 |

---

|  |  |
| --- | --- |
| Swiss-Prot ID | JAK2\_HUMAN |
|  | JAK2\_MOUSE |
|  | JAK2\_RAT |

---

|  |  |
| --- | --- |
| Cell Localization | Endomembrane system |
|  | Cytoplasm |
|  | Nucleus |

---

|  |  |
| --- | --- |
| Ensembl ID | ENSG00000096968 |
|  | ENSP00000371067.3 |
|  | ENST00000381652.3 |
|  | ENSMUSG00000024789 |
|  | ENSMUSP00000025705.6 |
|  | ENSMUST00000025705.6 |
|  | ENSMUSP00000064394.3 |
|  | ENSMUST00000065796.9 |
|  | ENSMUSP00000158405.1 |
|  | ENSMUST00000236990.1 |
|  | ENSRNOG00000059968 |
|  | ENSRNOP00000071877.1 |
|  | ENSRNOT00000087011.1 |
|  | ENSMUST00000025705 |
|  | ENSMUST00000065796 |
|  | ENST00000381652 |
|  | ENSRNOP00000071877 |
|  | ENSMUSP00000025705 |
|  | ENSRNOT00000087011 |
|  | ENSP00000371067 |
|  | ENSRNOG00000015547 |
|  | ENSMUSP00000064394 |
|  | ENSRNOP00000021218 |
|  | ENSRNOT00000021217 |

---

|  |  |
| --- | --- |
| MGI ID | MGI:96629 |
|  | 96629 |
|  | 2147795 |
|  | 2147569 |

---

|  |  |
| --- | --- |
| RGD ID | 2939 |

---

|  |  |
| --- | --- |
| Unigene ID | Rn.18909 |
|  | Mm.275839 |
|  | Hs.656213 |
|  | Hs.591081 |
|  | Mm.393199 |
|  | Hs.434374 |

---

|  |  |
| --- | --- |
| Homologene ID | 21033 |

---

|  |  |
| --- | --- |
| Shape | Sickle-vertex |

---

|  |  |
| --- | --- |
| IPI ID | IPI00207182 |
|  | IPI00031016 |
|  | IPI00122143 |
|  | IPI00885005 |
|  | IPI00476692 |

---

|  |  |
| --- | --- |
| Homo sapiens Chromosome position | 9p24 |

---

|  |  |
| --- | --- |
| Ensembl Transcript ID | ENST00000381652 |

---

|  |  |
| --- | --- |
| ENSEMBL | ENSG00000096968 |

---

|  |  |
| --- | --- |
| FunctionalClass | kinase |
|  | protein kinase |
|  | protein binding |
|  | protein-tyrosine kinase |
|  | nucleotide binding |
|  | SH2 domain binding |
|  | protein serine/threonine kinase |
|  | transferase |
|  | ATP binding |
|  | Janus kinase |
|  | receptor binding |
|  | growth hormone receptor binding |

---

|  |  |
| --- | --- |
| Mus musculus Chromosome position | 19 |
|  | 19 24.0 cM |

---

|  |  |
| --- | --- |
| KEGG pathway | Jak-STAT signaling pathway |
|  | Pancreatic cancer |
|  | Adipocytokine signaling pathway |

---

|  |  |
| --- | --- |
| Rattus norvegicus Chromosome position | 1q51-q53 |

---

|  |  |
| --- | --- |
| Ensembl Gene ID | ENSG00000096968 |

---

|  |  |
| --- | --- |
| ProteinAtlas NormalTissue | Tissue: adrenal gland; Cell type: glandular cells; Level: Moderate; Expression type: Staining; Reliability: Supportive |
|  | Tissue: cervix |
|  | uterine; Cell type: squamous epithelial cells; Level: Negative; Expression type: Staining; Reliability: Supportive |
|  | Tissue: tonsil; Cell type: non-germinal center cells; Level: Strong; Expression type: Staining; Reliability: Supportive |
|  | Tissue: oral mucosa; Cell type: squamous epithelial cells; Level: Weak; Expression type: Staining; Reliability: Supportive |
|  | Tissue: skeletal muscle; Cell type: myocytes; Level: Negative; Expression type: Staining; Reliability: Supportive |
|  | Tissue: kidney; Cell type: cells in tubules; Level: Strong; Expression type: Staining; Reliability: Supportive |
|  | Tissue: ovary; Cell type: follicle cells; Level: Weak; Expression type: Staining; Reliability: Supportive |
|  | uterine; Cell type: glandular cells; Level: Negative; Expression type: Staining; Reliability: Supportive |
|  | Tissue: spleen; Cell type: cells in red pulp; Level: Moderate; Expression type: Staining; Reliability: Supportive |
|  | Tissue: smooth muscle; Cell type: smooth muscle cells; Level: Weak; Expression type: Staining; Reliability: Supportive |
|  | Tissue: seminal vesicle; Cell type: glandular cells; Level: Strong; Expression type: Staining; Reliability: Supportive |
|  | Tissue: appendix; Cell type: glandular cells; Level: Moderate; Expression type: Staining; Reliability: Supportive |
|  | Tissue: appendix; Cell type: lymphoid tissue; Level: Moderate; Expression type: Staining; Reliability: Supportive |
|  | Tissue: bone marrow; Cell type: hematopoietic cells; Level: Moderate; Expression type: Staining; Reliability: Supportive |
|  | Tissue: breast; Cell type: glandular cells; Level: Weak; Expression type: Staining; Reliability: Supportive |
|  | Tissue: bronchus; Cell type: respiratory epithelial cells; Level: Moderate; Expression type: Staining; Reliability: Supportive |
|  | Tissue: cerebellum; Cell type: cells in granular layer; Level: Negative; Expression type: Staining; Reliability: Supportive |
|  | Tissue: cerebellum; Cell type: cells in molecular layer; Level: Weak; Expression type: Staining; Reliability: Supportive |
|  | Tissue: cerebellum; Cell type: Purkinje cells; Level: Moderate; Expression type: Staining; Reliability: Supportive |
|  | Tissue: cerebral cortex; Cell type: glial cells; Level: Negative; Expression type: Staining; Reliability: Supportive |
|  | Tissue: cerebral cortex; Cell type: neuronal cells; Level: Weak; Expression type: Staining; Reliability: Supportive |
|  | Tissue: cervix, uterine; Cell type: glandular cells; Level: Negative; Expression type: Staining; Reliability: Supportive |
|  | Tissue: cervix, uterine; Cell type: squamous epithelial cells; Level: Negative; Expression type: Staining; Reliability: Supportive |
|  | Tissue: colon; Cell type: glandular cells; Level: Moderate; Expression type: Staining; Reliability: Supportive |
|  | Tissue: duodenum; Cell type: glandular cells; Level: Strong; Expression type: Staining; Reliability: Supportive |
|  | Tissue: epididymis; Cell type: glandular cells; Level: Weak; Expression type: Staining; Reliability: Supportive |
|  | Tissue: esophagus; Cell type: squamous epithelial cells; Level: Negative; Expression type: Staining; Reliability: Supportive |
|  | Tissue: fallopian tube; Cell type: glandular cells; Level: Weak; Expression type: Staining; Reliability: Supportive |
|  | Tissue: gall bladder; Cell type: glandular cells; Level: Strong; Expression type: Staining; Reliability: Supportive |
|  | Tissue: heart muscle; Cell type: myocytes; Level: Weak; Expression type: Staining; Reliability: Supportive |
|  | Tissue: hippocampus; Cell type: glial cells; Level: Negative; Expression type: Staining; Reliability: Supportive |
|  | Tissue: hippocampus; Cell type: neuronal cells; Level: Weak; Expression type: Staining; Reliability: Supportive |
|  | Tissue: kidney; Cell type: cells in glomeruli; Level: Strong; Expression type: Staining; Reliability: Supportive |
|  | Tissue: lateral ventricle; Cell type: glial cells; Level: Negative; Expression type: Staining; Reliability: Supportive |
|  | Tissue: lateral ventricle; Cell type: neuronal cells; Level: Negative; Expression type: Staining; Reliability: Supportive |
|  | Tissue: liver; Cell type: bile duct cells; Level: Negative; Expression type: Staining; Reliability: Supportive |
|  | Tissue: liver; Cell type: hepatocytes; Level: Strong; Expression type: Staining; Reliability: Supportive |
|  | Tissue: lung; Cell type: macrophages; Level: Strong; Expression type: Staining; Reliability: Supportive |
|  | Tissue: lung; Cell type: pneumocytes; Level: Weak; Expression type: Staining; Reliability: Supportive |
|  | Tissue: lymph node; Cell type: germinal center cells; Level: Moderate; Expression type: Staining; Reliability: Supportive |
|  | Tissue: lymph node; Cell type: non-germinal center cells; Level: Strong; Expression type: Staining; Reliability: Supportive |
|  | Tissue: nasopharynx; Cell type: respiratory epithelial cells; Level: Weak; Expression type: Staining; Reliability: Supportive |
|  | Tissue: ovary; Cell type: ovarian stroma cells; Level: Negative; Expression type: Staining; Reliability: Supportive |
|  | Tissue: pancreas; Cell type: exocrine glandular cells; Level: Weak; Expression type: Staining; Reliability: Supportive |
|  | Tissue: pancreas; Cell type: islets of Langerhans; Level: Negative; Expression type: Staining; Reliability: Supportive |
|  | Tissue: parathyroid gland; Cell type: glandular cells; Level: Strong; Expression type: Staining; Reliability: Supportive |
|  | Tissue: placenta; Cell type: decidual cells; Level: Weak; Expression type: Staining; Reliability: Supportive |
|  | Tissue: placenta; Cell type: trophoblastic cells; Level: Weak; Expression type: Staining; Reliability: Supportive |
|  | Tissue: prostate; Cell type: glandular cells; Level: Negative; Expression type: Staining; Reliability: Supportive |
|  | Tissue: rectum; Cell type: glandular cells; Level: Moderate; Expression type: Staining; Reliability: Supportive |
|  | Tissue: salivary gland; Cell type: glandular cells; Level: Weak; Expression type: Staining; Reliability: Supportive |
|  | Tissue: skin; Cell type: epidermal cells; Level: Negative; Expression type: Staining; Reliability: Supportive |
|  | Tissue: small intestine; Cell type: glandular cells; Level: Strong; Expression type: Staining; Reliability: Supportive |
|  | Tissue: spleen; Cell type: cells in white pulp; Level: Moderate; Expression type: Staining; Reliability: Supportive |
|  | Tissue: stomach, lower; Cell type: glandular cells; Level: Weak; Expression type: Staining; Reliability: Supportive |
|  | Tissue: stomach, upper; Cell type: glandular cells; Level: Weak; Expression type: Staining; Reliability: Supportive |
|  | Tissue: testis; Cell type: cells in seminiferus ducts; Level: Moderate; Expression type: Staining; Reliability: Supportive |
|  | Tissue: testis; Cell type: Leydig cells; Level: Moderate; Expression type: Staining; Reliability: Supportive |
|  | Tissue: thyroid gland; Cell type: glandular cells; Level: Moderate; Expression type: Staining; Reliability: Supportive |
|  | Tissue: tonsil; Cell type: germinal center cells; Level: Weak; Expression type: Staining; Reliability: Supportive |
|  | Tissue: tonsil; Cell type: squamous epithelial cells; Level: Moderate; Expression type: Staining; Reliability: Supportive |
|  | Tissue: urinary bladder; Cell type: urothelial cells; Level: Weak; Expression type: Staining; Reliability: Supportive |
|  | Tissue: uterus, post-menopause; Cell type: cells in endometrial stroma; Level: Negative; Expression type: Staining; Reliability: Supportive |
|  | Tissue: uterus, post-menopause; Cell type: glandular cells; Level: Weak; Expression type: Staining; Reliability: Supportive |
|  | Tissue: uterus, pre-menopause; Cell type: cells in endometrial stroma; Level: Weak; Expression type: Staining; Reliability: Supportive |
|  | Tissue: uterus, pre-menopause; Cell type: glandular cells; Level: Weak; Expression type: Staining; Reliability: Supportive |
|  | Tissue: vagina; Cell type: squamous epithelial cells; Level: Negative; Expression type: Staining; Reliability: Supportive |
|  | Tissue: vulva/anal skin; Cell type: epidermal cells; Level: Negative; Expression type: Staining; Reliability: Supportive |

---

|  |  |
| --- | --- |
| LocatorString | pmftq:achondroplasia |

---
